# Regulation of surrogacy in India: whenceforth now?

**DOI:** 10.1136/bmjgh-2018-000986

**Published:** 2018-10-08

**Authors:** Bronwyn Parry, Rakhi Ghoshal

**Affiliations:** Global Health and Social Medicine, King’s College London, London, UK

**Keywords:** commercial gestational surrogacy, reproductive labour, assisted reproduction, regulation

## Background

Commercial gestational surrogacy (CGS) is unique among assisted reproductive services in its ability to attract disapprobation. Allied practices such as in vitro fertilisation (IVF), oocyte and sperm donation also enable infertile couples to reproduce through assistive technological and third-party interventions, yet they rarely attract sustained condemnation. Censure arises from concerns that CGS involves the routine exploitation of vulnerable, impoverished women of colour who are commissioned by wealthy white Westerners to perform a labour they would otherwise decline. Rationales for undertaking CGS are, however, multiple and complex.^1^ CGS is, moreover, increasingly used by citizens of the global south (often relatively impoverished ones) to address forms of structural infertility brought about by diseases such as genital tuberculosis.^2^ This intra-South demand, combined with the displacement of the fertility industry to emergent southern economies such as Thailand, Mexico and India, should serve to make the regulation of CGS a key concern for global reproductive health.

The legalisation of CGS in India in 2002^3^ led to increased demand from individuals, such as women with serious reproductive abnormalities and small numbers of male homosexual couples who are unable to biologically gestate their own children, and the market grew consequentially. However, in 2015 the Indian state, stung by critiques that CGS was inherently imperialist and oppressive, elected to ban all foreigners from accessing these services.^4^ Responding to further adverse publicity surrounding high-profile international cases of abandonment of surrogate children,^5^ a *Surrogacy (Regulation) Bill* was abruptly introduced in August 2016 that banned CGS altogether.^6^ Believing the key problem to lie in the ‘commercial’ element of the process, the Indian state determined to make surrogacy permissible only if performed altruistically. This, according to the Bill, would ‘prohibit the potential exploitation of surrogate mothers.’^7^ The problem, it would seem, was solved … or is it?

Allowing legislation to gestate out of an environment of moral panic driven by prominent cases, irrespective of their typicality or evidential basis, can result in changes that simply substitute one set of wrongs for another. Such, we argue, has been the case with this new regulation. In attempting to shut down what it perceives to be a degrading industry, the Bill dictates that only Indian women aged 25–35 years, with at least one existing child, and who are *closely related* to the intending couple are eligible to perform a surrogacy altruistically on their behalf. Any payment, reward or monetary incentive to the surrogate, her dependents or representatives is expressly forbidden; she may only receive medical expenses and insurance costs. Criminal penalties of ‘not less than three years and fines which may extend to five lakh rupees [half a million USD]’ for the doctor/clinic^8^ indicate the severity of the punishments for contravention.

## Ethical inconsistencies and contradictions

The basic presumption of the Bill, that banning commercial surrogacy will necessarily erase its exploitative potential is, we argue, highly problematic as it dismisses the possibility that altruistic arrangements can be equally as exploitative as commercial ones, although in different ways. The legal requirements for becoming an altruistic surrogate are now so prescriptive that they prove highly discriminatory for couples who would fail to access such a ‘close relative’.^i^ As a Delhi-based Assisted Reproductive Technologies (ART) specialist, offering surrogacy services explained:^ii^ ‘They [ie, the Bill] are saying you have to get a close relative; such as a sister. Now, say I do not have any sister. I have nobody [who fits the requirements]; then I can’t have a child.’ As she went on to argue: ‘If you feel that surrogacy is going to damage a woman, why are you then allowing the poor relative [to become a surrogate]?’

We agree that the State’s position here is ethically inconsistent. Reports occasionally emerge of instances in which a sister or even a mother becomes a surrogate (for her own grandchild), but these are relatively rare. As Mukerjee’s^9^ research reveals, very few women in India would voluntarily undertake surrogacy for someone within the family. It is imaginable therefore that such relatives, if found, could come under intense familial pressure to take up the role – especially if, for example, they are the daughters-in-law. We argue, therefore, that it is a gross error of judgement to assume that exploitation is essentially an economic problem, or that familial relations are devoid of exploitative or coercive potentials.

Prior regulations (viz, the ART (Regulation) Bill, 2010) advocated surrogate anonymity (her name does not appear on the birth certificate) and discouraged breast feeding to reduce postpartum bonding and promote separation after birth.^10^ The Surrogacy (Regulation) Bill, 2016, however, inexplicably abandons these arguments, allowing the commissioning parents to bring up a child born out of surrogacy in close proximity to the surrogate. We can only speculate on what toll this might take on the emotional health of the surrogate, the social mother and the child, but it seems reasonable to suggest that considerable psychological maladjustment could result, particularly if the true relationship between the surrogate and child is deliberately obscured or disavowed. Should a close relative agree (or be coerced) to become the surrogate but later change her mind, it would presumably also prove extremely difficult for her to terminate the pregnancy (for whatever reason, and especially when not medically indicated) without permanently damaging familial relations. These dynamics, we suggest, are arguably more socially and psychologically exploitative of vulnerable participants than those found in transparent contractual commercial relationships between consenting parties.

Few States legislate against payment for expended labour, but in this case the government is advocating that the surrogate receives no income for undertaking the pregnancy even if she left paid employment to do so – which we find highly discriminatory. The statement contained in the Bill that any women found undertaking a compensated surrogacy will be presumed to have been compelled to do so by ‘her husband, the intending couple or any other relative’^11^ fails to account for the woman’s own agency. As the aforementioned doctor notes: ‘Have you gone and checked what they [the poor women who are engaged in small-scale informal sector industries, and who often volunteer as commercial surrogates] are actually doing? Working in a windowless room, go and see how many hours you will survive there where they are working daily. The factories are windowless, and they are making all kinds of stuff … their children are loitering around for 10, 12, 14 hours. You think she would not prefer surrogacy to that?’ The presumptions made by the Bill are certainly highly patriarchal, as they imply that adult, mentally stable, although economically disadvantaged women lack the autonomy and decision-making capacity to voluntarily elect to undertake CGS as a viable alternative form of paid employment.

## Conclusion

In August 2017, the 102nd Indian Parliamentary Standing Committee^12^ critically engaged with the Bill and the Committee’s critique aligns with the assessments we offer here. Our joint analyses suggest that this legislation still requires significant further refinement. The role of legislation must be to assure high standards of care, consent and compensation for all involved in the delivery of reproductive services in India. This includes prospective surrogates and commissioning couples suffering the ignominy of infertility that arises from intractable reproductive disease. Some conditions such as uterine abnormality or absence, for which surrogacy is the only remedy, are immediately evident on medical examination. The Bill’s recommended minimum 5-year waiting period before accessing surrogacy thus has no scientific basis and is both arbitrary and discriminatory.

Indian (or other) citizens who are unable to bear biologically related children should have the same right to access commercial surrogacy as another form of assisted reproductive service, as do those whose infertility can be addressed via commercial IVF or compensated gamete provision. Surrogacy is but one of a suite of assisted reproductive services all of which require a consistent legislative approach. Economic disadvantage can compel poor women to engage in surrogacy as a form of employment, however this employment need not be inherently exploitative. The current absence of regulatory oversight and lack of legal protections for commercial surrogates is the root cause of exploitation. We conclude therefore, that there is no sensible rationale for sectioning the regulation of surrogacy out of the pre-existing ART (Regulation) Bill that has been languishing without legal ratification for many years now. The re-embedding of sensibly designed regulations within a revised ART Bill could enable a small number of accredited clinics to offer compensated surrogacy services under the strict oversight and regulation of a body akin to the UK’s Human Fertilisation and Embryology Authority. This could provide what all stakeholders most desire and need: a legislative solution that is nuanced, robust and, most importantly, non-discriminatory.

## Footnotes

A further shortcoming of the Bill is that it offers no definition of who constitutes ‘a close relative’.

Our Wellcome Trust funded qualitative research was designed to understand, using in-depth interviews and non-participant observation, how and in what ways cultural expectations, conventional practices and beliefs shape motivations to access ART services in India. By mapping the spread of ART from urban to non-urban markets in India we have also charted the political economy of this market and the ethical issues this expansion invokes. These are discussed at length in forthcoming research papers. We are indebted to all those who stakeholders we interviewed for this reasearch (over 120).
